# Supplementary material for: Density and diversity of macroinvertebrates in Colombian Andean streams impacted by mining, agriculture and cattle production
Source: PeerJ. 2020 Sep 16;8:e9619. doi: 10.7717/peerj.9619 (PMC7501782; doi:10.7717/peerj.9619)
Supplement: Table S1 — The HPCB parameters were measured between one and three times (n) per sampling zone, thus the value of each parameters per sampling showed as mean ± SD. [file peerj-08-9619-s001.docx]

| **Parameter** | **n** | **Reference** 1 | **Reference 2** | **Cattle production** | **Agriculture** | **Mining** |
| --- | --- | --- | --- | --- | --- | --- |
| Chemical oxygen demand (mg/L) | 3 | 23.3±14.4 | 29.67±10.21 | 25.3±17.9 | 50.3±58.6 | 20.3±6.8 |
| Biochemical oxygen demand (mg/L) | 3 | 3.2±0 | 3.21±5.44 | 3.21±0 | 3.2±0 | 3.2±0 |
| Total dissolved solids (mg/L) | 3 | 110.7±58.6 | 124±18.33 | 110.7±32.6 | 310.7±209.8 | 394.7±210 |
| Total suspended solids (mg/L) | 3 | 63.2±87.4 | 15.25±9.73 | 102.7±167.4 | 69.7±62 | 56.4±79.3 |
| Ammoniacal nitrogen (mg/L) | 2 | 0.1±0.08 | 0.05±0.04 | 0.1±0.06 | 0.2±0.2 | 0.1±0.1 |
| Nitrites (mg/L) | 3 | 0.07±0 | 0.07±0 | 0.07±0 | 0.1±0.03 | 0.3±0.2 |
| Sulphates (mg/L) | 3 | 21±1 | 11.33±0.58 | 6.7±0.6 | 9.3±3.5 | 55.7±30.5 |
| Fe (mg/L) | 3 | 0.4±0.4 | 0.06±0.02 | 0.2±0.06 | 0.8±0.9 | 1.3±0.9 |
| Chlorides (mg/L) | 3 | 2.5±0 | 2.5±0 | 2.5±0 | 3.2±0.8 | 2.9±0.6 |
| Phosphates (mg/L) | 3 | 0.7±0.62 | 0.3±0.17 | 0.4±0.2 | 0.4±0.3 | 1.2±0.62 |
| Cyanide (mg/L) | 1 | *0.001 | *0.001 | *0.001 | *0.001 | *0.001 |
| Hg (mg/L) | 3 | *0.2 | *0.2 | *0.2 | *0.2 | *0.2 |
| Al (mg/L) | 1 | *7.3±0.3 | *7.3±0.3 | *7.3±0.3 | *7.810.3 | *7.4±0.3 |
| Pb (mg/L) | 2 | *0.02±0.01 | *0.02±0.01 | *0.02±0.01 | *0.02±0.01 | *0.02±0.01 |
| B (mg/L) | 2 | *1.1±0.5 | *1.1±0.5 | *1.1±0.5 | *1.1±1.1 | *1.1±0.5 |
| Fats and oils (mg/L) | 3 | 0.4±0.2 | 0.83±0.58 | 0.8±0.6 | 0.4±0.2 | 0.9±0.7 |
| Dissolved oxygen (mg/L) | 3 | 9.3±3.3 | 4.75±2.64 | 5.4±0.63 | 2.3±0.8 | 5.2±0.6 |
| pH | 3 | 7.6±0.3 | 8.27±0.08 | 7.5±0.07 | 7.7±0.09 | 7.7±0.2 |
| Temperature (ºC) | 3 | 12.1±0.4 | 18.9±0.48 | 13.5±1.15 | 17.9±0.7 | 13.1±0.05 |
| Conductivity (µS/m) | 3 | 108±9 | 99.675±4.35 | 139±86 | 33.8±25.5 | 131.5±55.5 |
| Total coliforms (CFU/100mL) | 2 | 2375 | 2200 | 806 | 209650 | 2933.5 |
| *Escherichia coli* (CFU/100mL) | 2 | 12.4±9.7 | 49 | 65.9 | 90.95 | 6.3 |
| Depth (cm) | 3 | 9.2±3.8 | 18.2±4.9 | 10.8±4.02 | 7.2±2.2 | 9.5±2.7 |
| Width (m) | 3 | 1.8±0.2 | 1.82±0.49 | 1.7±0.6 | 1.6±0.4 | 1.9±0.6 |
| Flow (m/s) | 3 | 0.4±0.09 |  | 0.4±0.05 | 0.2±0.05 | 0.4±0.2 |

*Below the detection limit.
