# Supplementary material for: Density and diversity of macroinvertebrates in Colombian Andean streams impacted by mining, agriculture and cattle production
Source: PeerJ. 2020 Sep 16;8:e9619. doi: 10.7717/peerj.9619 (PMC7501782; doi:10.7717/peerj.9619)
Supplement: Table S2 [file peerj-08-9619-s002.docx]

| Sampling zone | Variable | Sampling time | | | | | |
| --- | --- | --- | --- | --- | --- | --- | --- |
|  |  | 2014-02-12 | 2014-04-01 | 2014-07-08 | 2014-09-09 | 2014-11-04 | 2015-02-10 |
| Reference 1 | Flow volume | 0.16 | 0.04 | 0.03 | 0.02 | 0.05 | 0.08 |
|  | Density | 253.09 | 150.62 | 356.79 | 401.23 | 425.93 | 290.12 |
| Reference 2 | Flow volume | 0.05 | 0.04 | 0.21 | 0.02 | 0.03 | 0.07 |
|  | Density | 328.40 | 237.04 | 845.68 | 633.33 | 292.59 | 435.80 |
| Cattle production | Flow volume | 0.22 | 0.06 | 0.07 | 0.04 | 0.06 | 0.09 |
|  | Density | 244.44 | 254.32 | 345.68 | 165.43 | 277.78 | 406.17 |
| Agriculture | Flow volume | 0.02 | 0.01 | 0.04 | 0.01 | 0.01 | 0.02 |
|  | Density | 685.19 | 64.20 | 262.96 | 133.33 | 58.02 | 104.94 |
| Mining | Flow volume | 0.14 | 0.11 | 0.26 | 0.06 | 0.07 | 0.13 |
|  | Density | 230.86 | 81.48 | 414.81 | 543.21 | 223.46 | 143.21 |
|  | Precipitation | 7.70 | 0.20 | 0.98 | 0.04 | 7.88 | 5.04 |
