# Supplementary figures and images for: Density and diversity of macroinvertebrates in Colombian Andean streams impacted by mining, agriculture and cattle production
Source: PeerJ. 2020 Sep 16;8:e9619. doi: 10.7717/peerj.9619 (PMC7501782; doi:10.7717/peerj.9619)

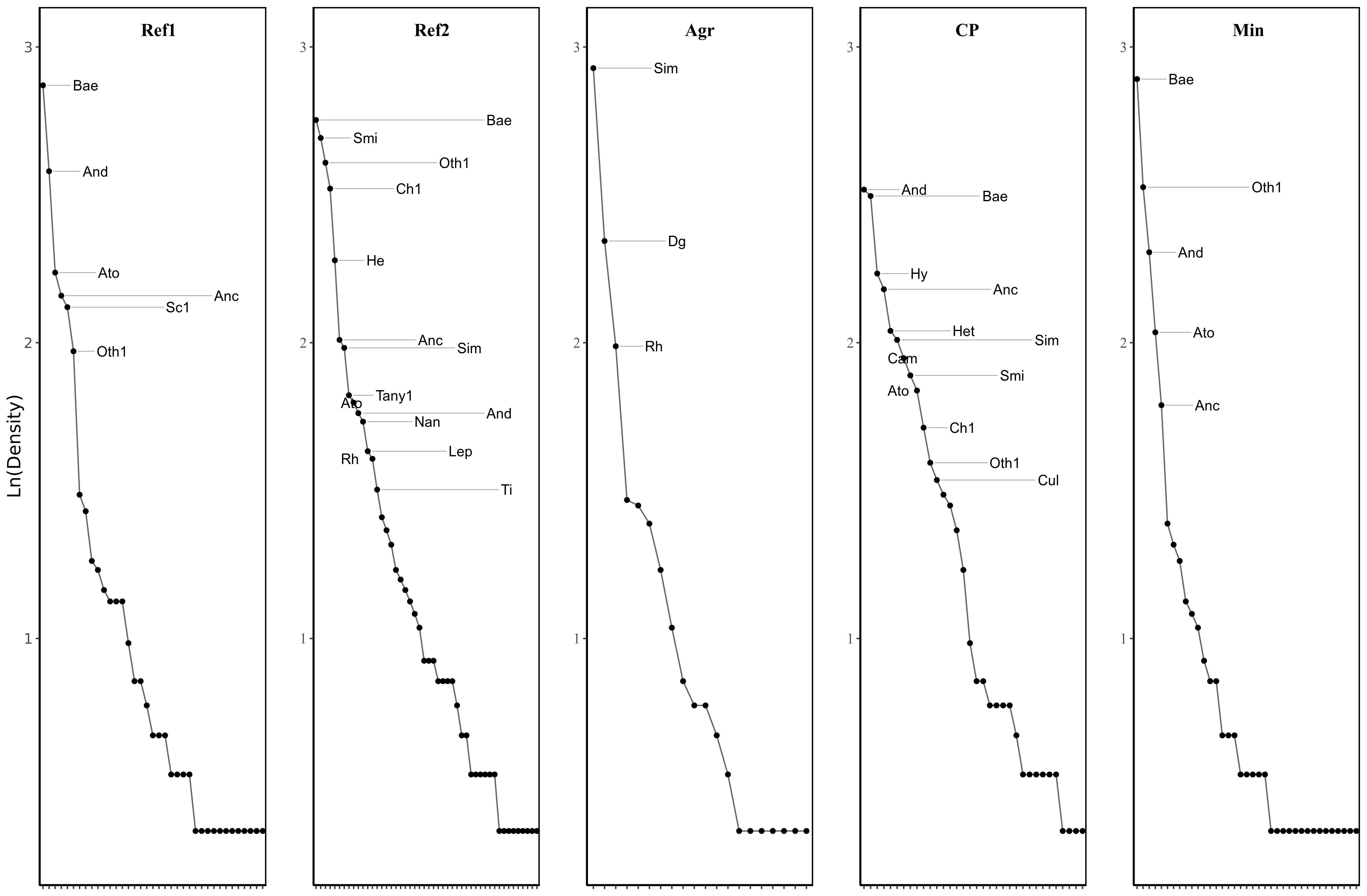

Supplement: Data S1 [file peerj-08-9619-s004.zip › Data_S1/2_RankD_curve/Figure_3.jpeg]

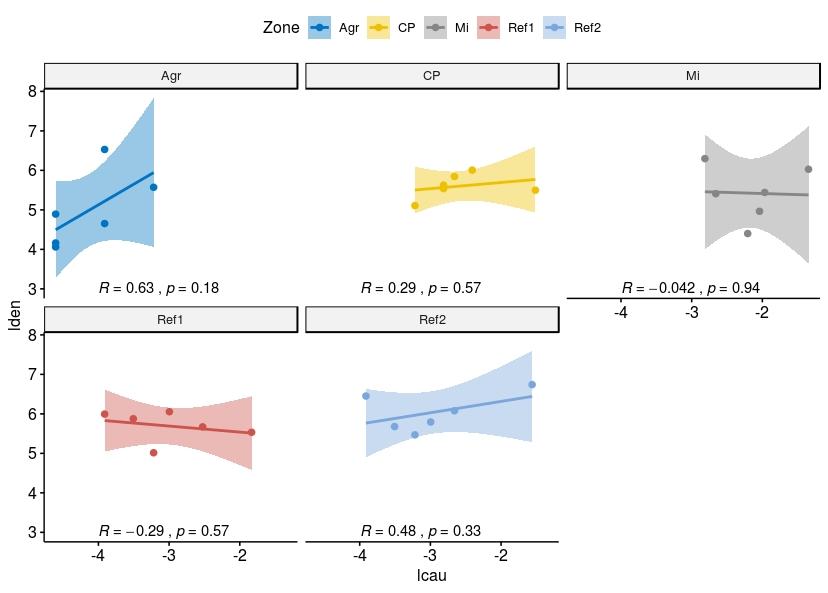

Supplement: Figure S1 — Ref1 = Reference 1; Ref2 = Reference 2; Agr = Agriculture; CP = Cattle production; Mi = Mining; Icau = water flow (ln); Iden = RTUs density (ln). [file peerj-08-9619-s005.docx]

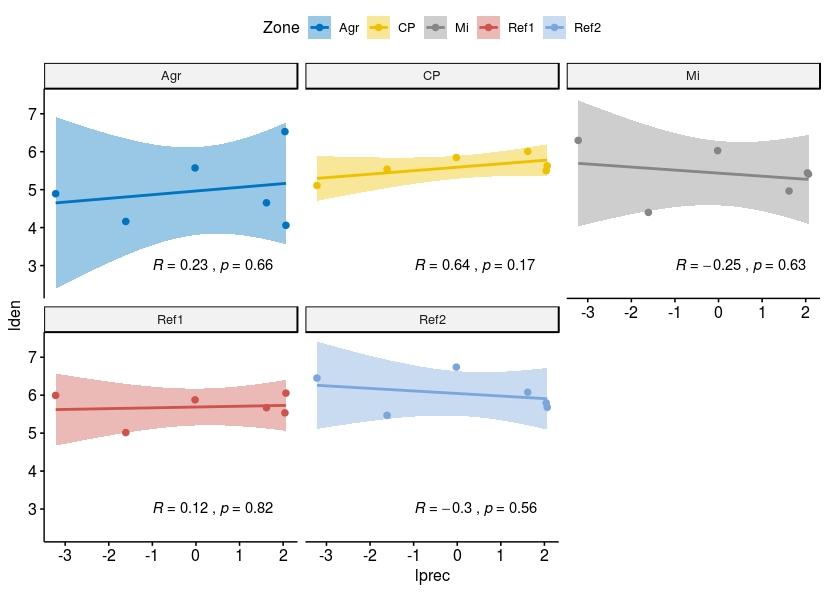

Supplement: Figure S2 — Ref1 =Reference 1; Ref2 =Reference 2; Agr =Agriculture; CP =Cattle production; Mi =Mining; Ipre = (ln); Iden = RTUs density (ln). [file peerj-08-9619-s006.docx]
